# Supplementary material for: Hedgehog-stimulated phosphorylation at multiple sites activates Ci by altering Ci–Ci interfaces without full Suppressor of Fused dissociation
Source: PLoS Biol. 2025 Apr 11;23(4):e3003105. doi: 10.1371/journal.pbio.3003105 (PMC12052134; doi:10.1371/journal.pbio.3003105)
Supplement: S1 Fig — (A) Key Ci features are illustrated. The top cartoon shows Su(fu) binding regions (red), the zinc finger domain (ZF, which binds DNA and can bind Cos2), and the binding region for CBP co-activator. The second cartoon shows deletions employed in this study (pink, yellow, and blue) and the CORD Cos2 binding domain. The third cartoon shows Fu phosphorylation sites examined in this study. PKA sites (S838, S856, S892) that promote Ci-155 processing, and a third Cos2 binding region (CDN; 346–440) are not shown. (B–H) Third instar wing discs (20× objective for (B) and 63× objective for all other images) with one copy of the indicated ci CRISPR allele, GFP marking homozygous cos2 mutant clones (green; yellow arrowheads), and yellow dotted lines marking the AP border. (B’–H’) Ptc-lacZ expression (red) and (B’’–H’’) En protein (gray-scale) in the same discs. In (B) the entire wing, disc lacks Su(fu) activity. Scale bars are 100 μm for (B) and 40 μm for all other images. (DOCX) [file pbio.3003105.s002.docx]

**S1 Fig (Related to Fig. 1). Loss of Su(fu) binding sites or two SYGHI-adjacent regions increase Ci activity in *cos2* mutant clones: effects on En induction.**

(**A)** Key Ci features are illustrated. The top cartoon shows Su(fu) binding regions (red), the zinc finger domain (ZF, which binds DNA and can bind Cos2) and the binding region for CBP co-activator. The second cartoon shows deletions employed in this study (pink, yellow and blue) and the CORD Cos2 binding domain. The third cartoon shows Fu phosphorylation sites examined in this study. PKA sites (S838, S856, S892) that promote Ci-155 processing, and a third Cos2 binding region (CDN; 346-440) are not shown. (**B-H**) Third instar wing discs (20x objective for (**B**) and 63x objective for all other images) with one copy of the indicated *ci* CRISPR allele, GFP marking homozygous *cos2* mutant clones (green; yellow arrowheads), and yellow dotted lines marking the AP border. (**B’-H‘**) Ptc-lacZ expression (red) and (**B’’-H’’**) En protein (gray-scale) in the same discs. In (**B**) the entire wing disc lacks Su(fu) activity. Scale bars are 100μm for (**B**) and 40μm for all other images.
