# Supplementary material for: Hedgehog-stimulated phosphorylation at multiple sites activates Ci by altering Ci–Ci interfaces without full Suppressor of Fused dissociation
Source: PLoS Biol. 2025 Apr 11;23(4):e3003105. doi: 10.1371/journal.pbio.3003105 (PMC12052134; doi:10.1371/journal.pbio.3003105)
Supplement: S2 Fig — (A–C) Third instar wing discs (63× objective) with one copy of (A, C) Ci-WT-Sufu or (B) Ci-SYAAD-Sufu, GFP marking pka mutant clones (green; yellow arrowheads), and yellow dotted lines marking the AP border. (A’–C’) Ptc-lacZ expression (red) and (A”–C”) Ci-155 expression (gray-scale) in the same discs. (C) Su(fu) activity was absent in the whole disc. Scale bars are 40 μm. (D) Bar graph showing the average ratio of Ptc-lacZ intensity and Ci-155 intensity in clones relative to the AP border of wild-type control discs, together with SEMs (n values 94, 30, and 23, respectively, for each set of three genotypes). Differences with p < 0.005 (Student t test with Welch correction) are indicated for comparing to Ci-WT-Sufu in an otherwise wild-type disc (red asterisk). (E) Bar graph showing the average ratio of Ci-155 intensity in the indicated clones relative to the AP border of wild-type control discs, together with SEMs (n values 20, 5, 14, 10, 87, 19, 40, 54, 46, 47, 199, and 90); wing disc images and ptc-lacZ graph for these clones are in Fig 4. Differences with p < 0.005 (Student t test with Welch correction) are indicated for comparing a Ci variant to Ci-WT (black asterisk) or for comparisons between bracketed pairs (red asterisk). Please see Materials and methods for details of measurements and expression of all experimental values relative to AP border values of control wild-type wing discs. The data underlying the graphs shown in the figure can be found in S4 Data. (DOCX) [file pbio.3003105.s003.docx]

**S2 Fig (Related to Fig. 4). Su(fu) inhibition is increased by covalent linkage to Ci but still requires non-covalent binding to the SYGHI region of Ci: effects in PKA mutant clones.**

(**A-C**) Third instar wing discs (63x objective) with one copy of (**A, C**) Ci-WT-Sufu or (**B**) Ci-SYAAD-Sufu, GFP marking *pka* mutant clones (green; yellow arrowheads), and yellow dotted lines marking the AP border. (**A’-C’**) Ptc-lacZ expression (red) and (**A”-C”**) Ci-155 expression (gray-scale) in the same discs. (**C**) Su(fu) activity was absent in the whole disc. Scale bars are 40μm. (**D**) Bar graph showing the average ratio of Ptc-lacZ intensity and Ci-155 intensity in clones relative to the AP border of wild-type control discs, together with SEMs (n values 94, 30, and 23, respectively, for each set of three genotypes). Differences with p<0.005 (Student’s t test with Welch correction) are indicated for comparing to Ci-WT-Sufu in an otherwise wild-type disc (red asterisk). (**E**) Bar graph showing the average ratio of Ci-155 intensity in the indicated clones relative to the AP border of wild-type control discs, together with SEMs (n values 20, 5, 14, 10, 87, 19, 40, 54, 46, 47, 199, and 90); wing disc images and *ptc-lacZ* graph for these clones are in Fig. 4. Differences with p<0.005 (Student’s t test with Welch correction) are indicated for comparing a Ci variant to Ci-WT (black asterisk) or for comparisons between bracketed pairs (red asterisk). Please see Materials and Methods for details of measurements and expression of all experimental values relative to AP border values of control wild-type wing discs. The data underlying the graphs shown in the figure can be found in S4_data.
