# Supplementary material for: Hedgehog-stimulated phosphorylation at multiple sites activates Ci by altering Ci–Ci interfaces without full Suppressor of Fused dissociation
Source: PLoS Biol. 2025 Apr 11;23(4):e3003105. doi: 10.1371/journal.pbio.3003105 (PMC12052134; doi:10.1371/journal.pbio.3003105)
Supplement: S3 Fig — (A, B, F–M) Third instar wing discs (20× objective for (G) and 63× objective for other images) with the named Ci variant, GFP marking the indicated clone types (green; yellow arrowheads), and yellow dotted lines marking the AP border. (C–G) All have Ci ∆1,201–1,271, while (A, B) have GAP-Fu clones and (J-M) have cos2 GAP-Fu clones. (A’, B’, F’–M’) Ptc-lacZ expression (red) and (A”, B”, F”–H”, J”–M”) Ci-155 expression (gray-scale) in the same discs. (A, B, G, I–M) GAP-Fu and cos2 GAP-Fu clones also lack smo activity. (A, B) Both S218A (Ci-A1) and S1230A (Ci-A2) reduced Ptc-lacZ induction by GAP-Fu. (C–E) Third instar wing discs with one copy of Ci ∆1,201–1,271, showing Ptc-lacZ (red) and (C’, E’) Ci-155 expression (gray-scale) (20× objective) or (D’) En expression (green) (63× objective), resembling Ci-WT behavior, with the AP border marked by dotted yellow lines. (F, G) Induction of Ptc-lacZ and Ci-155 levels for Ci ∆1,201–1,271 also resembled Ci-WT in (F) cos2 and (G) GAP-Fu clones. (H–M) The addition of GAP-Fu in cos2 mutant clones (I’) increased Ptc-lacZ (compare to Fig 1E) and (I”) En induction by Ci-WT but decreased Ptc-lacZ induction by (H’, J’) Ci ΔΔ (which lacks residues 175–230 and 1,201–1,271) and by (K’–M’) Ci variants lacking residues 1,370–97, 175–230, or 270–300 (compare to Fig 1H, I, K). Scale bars are 40 µm for (A, B, F, H–M), 100 µm for (C, E, G), and 20 µm for (D). (DOCX) [file pbio.3003105.s004.docx]

**S3 Fig (Related to Figure 5). S218 and S1230 each contribute to activation by Fu and GAP-Fu reduces the activity of several Ci deletion variants in *cos2* mutant clones, as for Ci-A1A2.**

(**A, B, F-M**) Third instar wing discs (20x objective for (**G**) and 63x objective for other images) with the named Ci variant, GFP marking the indicated clone types (green; yellow arrowheads), and yellow dotted lines marking the AP border. (**C-G**) all have Ci Δ1201-1271, while (**A, B**) have *GAP-Fu* clones and (**J-M**) have *cos2 GAP-Fu* clones. (**A‘, B‘, F‘-M‘**) Ptc-lacZ expression (red) and (**A“, B“, F“-H“, J“-M“**) Ci-155 expression (gray-scale) in the same discs. (**A, B, G, I-M**) *GAP-Fu* and *cos2 GAP-Fu* clones also lack *smo* activity. (**A, B**) Both S218A (Ci-A1) and S1230A (Ci-A2) reduced Ptc-lacZ induction by GAP-Fu. (**C-E**) Third instar wing discs with one copy of Ci Δ1201-1271, showing Ptc-lacZ (red) and (**C’, E’**) Ci-155 expression (gray-scale) (20X objective) or (**D’**) En expression (green) (63X objective), resembling Ci-WT behavior, with the AP border marked by dotted yellow lines. (**F, G**) Induction of Ptc-lacZ and Ci-155 levels for Ci Δ1201-1271 also resembled Ci-WT in (**F**) *cos2* and (**G**) *GAP-Fu* clones. (**H-M**) The addition of GAP-Fu in *cos2* mutant clones (**I‘**) increased Ptc-lacZ (compare to Fig. 1E) and (**I”**) En induction by Ci-WT but decreased Ptc-lacZ induction by (**H‘, J‘**) Ci ΔΔ (which lacks residues 175-230 and 1201-1271) and by (**K’-M’**) Ci variants lacking residues 1370-97, 175-230 or 270-300 (compare to Fig. 1H, I, K). Scale bars are 40μm for (**A, B, F, H-M**), 100μm for (**C, E, G**) and 20 μm for (**D**).
