# Supplementary material for: Hedgehog-stimulated phosphorylation at multiple sites activates Ci by altering Ci–Ci interfaces without full Suppressor of Fused dissociation
Source: PLoS Biol. 2025 Apr 11;23(4):e3003105. doi: 10.1371/journal.pbio.3003105 (PMC12052134; doi:10.1371/journal.pbio.3003105)
Supplement: S4 Fig — (A, D, F, I) Third instar wing disc with one copy of Ci-A1A2 (crCi-A1A2/ci94), showing (A, D, F, I) Ptc-lacZ (red) and (A’, D’, F’, I’) Ci-155 expression (gray-scale) (20× objective). The discs are (A) otherwise wild-type or (D, F, I) Su(fu)LP/LP but expressing the indicated UAS-Su(fu) transgene using C765-GAL4. (B, E, G,J, M-O) Third instar wing disc with two copies of Ci-A1A2, showing (B, E, G, J, M, O) Ptc-lacZ (red) and (B’, E’, G’, J’, M’, O’) Ci-155 expression (gray-scale) (20× objective) or (C, H K, N) Ptc-lacZ (red) and (C’, H’, K’, N’) En expression (green) (63× objective; AP border marked by dotted yellow lines). The discs are (B, C) otherwise wild-type, (E, G, J) Su(fu)LP/LP but expressing the indicated UAS-Su(fu) transgene using C765-GAL4 or (M–O) additionally lack endogenous cos2 activity but contain one copy of the genomic transgene gCos-AA (encoding S572A S931A alterations). Su(fu)-5A has Fu site Ser residues substituted by Ala; mSufu encodes mouse Sufu. Scale bars are 20 μm for (C, H, K, N) and 100 μm for all other images. (L) Bar graph showing the ratio of Ptc-lacZ intensity at the AP border of the named genotypes (with Cos-AA replacement of endogenous Cos2 in pink) relative the AP border of wild-type discs, together with SEMs (n = 29, 16, 27, 20, 17, 3, 22, 36, 28, and 15, respectively, for wing discs with Ci-A1A2). The blue dotted line at 1.0 marks the Ci-WT value. Differences with p < 0.005 (Student t test with Welch correction) are indicated for comparing ptc-lacZ at the AP border for Ci-A1A2 together with the indicated Su(fu) variant, or additional Cos2 variant, to Ci-A1A2 alone (separately for either one copy or two copies of Ci-A1A2) (red asterisk). Please see Materials and methods for details of measurements and expression of all experimental values relative to AP border values of control wild-type wing discs. The data underlying the graphs shown in the figure can be found in S7 Data. (DOCX) [file pbio.3003105.s005.docx]

**S4 Fig (Related to Figure 7). Loss of Fu sites in Su(fu) and Cos2 do not further reduce activity of Ci lacking S218 and S1230 Fu sites.**

(**A, D, F, I**) Third instar wing disc with one copy of Ci-A1A2 (*crCi-A1A2/ci^94^*), showing (**A, D, F, I**) Ptc-lacZ (red) and (**A’, D’, F’, I’**) Ci-155 expression (gray-scale) (20X objective). The discs are (**A**) otherwise wild-type or (**D, F, I**) *Su(fu)^LP/LP^* but expressing the indicated *UAS-Su(fu)* transgene using *C765-GAL4.* (**B, E, G,J, M-O**) Third instar wing disc with two copies of Ci-A1A2, showing (**B, E, G, J, M, O**) Ptc-lacZ (red) and (**B’, E’, G’, J’, M’, O’**) Ci-155 expression (gray-scale) (20X objective) or (**C, H K, N**) Ptc-lacZ (red) and (**C’, H’, K’, N’**) En expression (green) (63X objective; AP border marked by dotted yellow lines). The discs are (**B, C**) otherwise wild-type, (**E, G, J**) *Su(fu)^LP/LP^* but expressing the indicated *UAS-Su(fu)* transgene using *C765-GAL4* or (**M-O**) additionally lack endogenous *cos2* activity but contain one copy of the genomic transgene *gCos-AA* (encoding S572A S931A alterations). Su(fu)-5A has Fu site Ser residues substituted by Ala; mSufu encodes mouse Sufu. Scale bars are 20μm for (**C, H, K, N**) and 100 μm for all other images. (**L**) Bar graph showing the ratio of Ptc-lacZ intensity at the AP border of the named genotypes (with Cos-AA replacement of endogenous Cos2 in pink) relative the AP border of wild-type discs, together with SEMs (n= 29, 16, 27, 20, 17, 3, 22, 36, 28, 15 respectively for wing discs with Ci-A1A2). The blue dotted line at 1.0 marks the Ci-WT value. Differences with p<0.005 (Student’s t test with Welch correction) are indicated for comparing *ptc-lacZ* at the AP border for Ci-A1A2 together with the indicated Su(fu) variant, or additional Cos2 variant, to Ci-A1A2 alone (separately for either one copy or two copies of Ci-A1A2) (red asterisk). Please see Materials and Methods for details of measurements and expression of all experimental values relative to AP border values of control wild-type wing discs. The data underlying the graphs shown in the figure can be found in S7_data.
