# Supplementary material for: Hedgehog-stimulated phosphorylation at multiple sites activates Ci by altering Ci–Ci interfaces without full Suppressor of Fused dissociation
Source: PLoS Biol. 2025 Apr 11;23(4):e3003105. doi: 10.1371/journal.pbio.3003105 (PMC12052134; doi:10.1371/journal.pbio.3003105)
Supplement: S5 Fig — (A–D, J–L) One copy of named Ci variants in (A, B, J, K) otherwise wild-type, (C, D) fumH63 Su(fu)LP/LP or (L) fumH63 third instar wing discs, showing (A–D, J–L) Ptc-lacZ (red), (A’, J’, L’) Ci-155 (gray-scale), or (B’–D’, K’) En expression (green, with the AP border marked by dotted yellow lines. (E–I) Third instar wing discs (63× objective) with one copy of (E) Ci-D1D2D3, with Ptc-lacZ (red) and En (green) expression shown in cos2 clones (yellow arrowheads), or with (F, G) Ci-A1A2A3 or (H, I) Ci-D1D2avA3, GFP marking (F-I) smo GAP-Fu clones (green; yellow arrowheads) in otherwise (F, H) wild-type or (G, I) Su(fu)LP/LP discs, and yellow dotted lines marking the AP border. Scale bars are 100 μm for (A, J, L), 20 μm for (B, C, D, K), and 40 μm for all other images. (M) Bar graph showing the ratio of Ptc-lacZ intensity at the AP border for the named Ci variants and Su(fu) genotypes relative to the AP border of control discs, together with SEMs (n = 20, 17, 15, 29, 22, 23, and 7, respectively). (N) Bar graph showing the ratio of Ptc-lacZ intensity at the AP border for the named Ci variants in otherwise wild-type (red) and fum63 wing discs (pink) relative to the AP border of control discs, together with SEMs (n = 29, 15, 24, 39, 13, 17, and 12, respectively, for wild-type and 38, 5, 7, 21, 7, 20, 14, and 10, respectively, for fumH63). The difference between pairs of values shows the contribution of Fu kinase activity at the AP border. (M and N) Differences with p < 0.005 (Student t test with Welch correction) are indicated for comparing a Ci variant to Ci-WT (black asterisk) or comparisons between bracketed pairs (red asterisk). Please see Materials and methods for details of measurements and expression of all experimental values relative to AP border values of control wild-type wing discs. The data underlying the graphs shown in the figure can be found in S8 Data. (DOCX) [file pbio.3003105.s006.docx]

**S5 Fig. (Related to Figure 8) Contributions of S286, T294, S1382 and S1385 to Ci activation.**

**(A-D, J-L**) One copy of named Ci variants in (**A, B, J, K**) otherwise wild-type, (**C, D**) *fu^mH63^ Su(fu)^LP/LP^* or (**L**) *fu^mH63^* third instar wing discs, showing (**A-D, J-L**) Ptc-lacZ (red), (**A’, J’, L’**) Ci-155 (gray-scale) or (**B’-D’, K’**) En expression (green, with the AP border marked by dotted yellow lines. (**E-I**) Third instar wing discs (63x objective) with one copy of (**E**) Ci-D1D2D3, with Ptc-lacZ (red) and En (green) expression shown in *cos2* clones (yellow arrowheads), or with (**F, G**) Ci-A1A2A3 or (**H, I**) Ci-D1D2avA3, GFP marking (**F-I**) *smo GAP-Fu* clones (green; yellow arrowheads) in otherwise (**F, H**) wild-type or (**G, I**) *Su(fu)^LP/LP^* discs, and yellow dotted lines marking the AP border. Scale bars are 100μm for (**A, J, L**), 20μm for (**B, C, D, K**) and 40μm for all other images. (**M**) Bar graph showing the ratio of Ptc-lacZ intensity at the AP border for the named Ci variants and Su(fu) genotypes relative to the AP border of control discs, together with SEMs (n= 20, 17, 15, 29, 22, 23, and 7, respectively). (**N**) Bar graph showing the ratio of Ptc-lacZ intensity at the AP border for the named Ci variants in otherwise wild-type (red) and *fu^m63^* wing discs (pink) relative to the AP border of control discs, together with SEMs (n= 29, 15, 24, 39, 13, 17, and 12, respectively for wild-type and 38, 5, 7, 21, 7, 20, 14, and 10, respectively, for *fu^mH63^*). The difference between pairs of values shows the contribution of Fu kinase activity at the AP border. (**M-N**) Differences with p<0.005 (Student’s t test with Welch correction) are indicated for comparing a Ci variant to Ci-WT (black asterisk) or comparisons between bracketed pairs (red asterisk). Please see Materials and Methods for details of measurements and expression of all experimental values relative to AP border values of control wild-type wing discs. The data underlying the graphs shown in the figure can be found in S8_data.
