# Supplementary material for: Hedgehog-stimulated phosphorylation at multiple sites activates Ci by altering Ci–Ci interfaces without full Suppressor of Fused dissociation
Source: PLoS Biol. 2025 Apr 11;23(4):e3003105. doi: 10.1371/journal.pbio.3003105 (PMC12052134; doi:10.1371/journal.pbio.3003105)
Supplement: Attachment — Submitted filename: HhFu revisions4.docx [file pbio.3003105.s017.docx]

We are very grateful for the thorough and balanced critical review of our manuscript by reviewers and editors, leading to numerous improvements, as detailed below. We believe that the sum of these responses meets the guidelines set out by the editor for publication of our revised submission. A significant set of changes clarified where primary data can be found, exactly how it was gathered and processed, including statistics and where relevant information can be found. Supporting files of spreadsheets for each figure are a significant component of full data exposition. We therefore specifically request (should the submission be accepted) that the organization and naming of these supplementary files is retained and/or that production staff consult with us on final appearance and names.

Reviewer 1

The manuscript by Kim et al. describes complex models for Ci regulation by Sufu, Fu, and Cos2 through analyzing the individual and combined effects of Fu phosphorylation residues in Ci.  The authors developed an impressive in vivo model to dissect the activity of various forms of Ci in inducing Ptc-lacZ expression in Drosophila wing disc. One model suggest that Ci-Ci interaction is regulated through Ci phosphorylation by Fu, although additional supporting materials are needed. By mutating a series of Fu phosphorylation sites in endogenous Ci, the authors sophisticatedly analyzed Ci activity under various conditions such as Fu mutation, Sufu mutation, and/or Cos2 mutation. The study provides extensive data and physiological relevance for the mechanisms of Ci regulation by multiple factors including Fu, Sufu, and Cos2, potentially offering a comprehensive understanding of Ci regulation in vivo. However, the manuscript contains several misleading descriptions that need to be
carefully addressed. Additional experiments are needed to address Ci-Ci interaction, while other concerns can be addressed by rewriting the statements and discussion. 

Major concerns:

We have added numbers to each point for clarity. The alterations have changed line numbers (partly because we did not embed figures & legends in the revised, tracked text). Hence, current line number references are added.

This manuscript needs editing for typos and confusing statements (some examples are listed below).

We responded to the specific examples as described below, and made additional changes.

1. Ci-Ci interaction model: The authors proposed an intriguing model for Ci-Ci interaction therefore activation independent of Sufu regulation; however, there is no further evidence to support this idea. Does Ci interact with itself in co-immune precipitation, co-localization, and/or in vitro interaction assays? Does Hh signaling regulate Ci-Ci interaction in this model? Additionally, does Ci-Ci interaction apply to Ci155-Ci75?

These are obviously important questions both for understanding detailed mechanism and for confirming the proposed general model. The candid answer to the main question has two components. First, we believe that only physiological data on Ci-Ci interactions would be truly relevant. There are no proven methods for examining Ci interactions of this type carefully under different conditions in Drosophila tissue. Second, it might be expected that we could use established techniques to look for potentially relevant Ci-Ci interactions using non-physiological conditions. We had planned to do that during our studies (even though we are skeptical of the relevance) but had no available capacity, choosing instead to make our genetic analyses as thorough as possible. That approach appeared to be the best value, given our unique positioning with regard to reagents, appetite and experience for genetic analyses and our relatively limited expertise in examining protein interactions. It was our hope that dissemination of the results of our extensive analyses and model would provide a sound foundation and inspiration for others to have the opportunity to investigate the details of protein-protein interactions. We are grateful for the editorial judgment, in line with that aspiration, that the strength of our genetic analyses suffices for a significant gain in understanding Hh signal transduction appropriate to the journal.

Certainly, Hh signaling, by way of Fu kinase activity, regulates Ci-Ci interaction in the model.

We do not know if there are interactions between Ci-155 and Ci-75. However, this seems unlikely to be of major relevance since (i) the relevant Ci-Ci interactions are within Ci-Su(fu) complexes and Ci-75 appears largely to be absent from Su(fu) complexes, and (ii) activation by Fu kinase is most relevant in locations where Ci-155 processing is substantially inhibited.

2. Figure 1F-F", needs larger clones. The changes in Ci level might not be visible in small clones.

We have replaced Fig. 1F-F” (now G-G”) with a disc containing larger clones. The result for Ci-155 levels is the same (very little excess in cos2 clones relative to Ci-WT), confirmed by quantitation in all discs.

3. Figure 1M&N, need statistical analysis to indicate the significance. SEM is insufficient to identify the significance among samples. This concern applies to all similar figures.

We have added statistical tests for significance of differences to these graphs and all others. We used Student’s unpaired two-tailed t-test with Welch correction for unequal sample numbers. In all cases, a variant Ci was compared to Ci-WT. In several cases, other specific pairs of Ci variants or changes in conditions for a specific Ci variant were compared (& shown in the figure). All of the results of comparisons are in the supplementary spreadsheets. In the graphs we decided to use the same conventions throughout the paper. First, we indicated p values less than 0.005 with an asterisk. We chose this value because the number of genotypes being compared altogether for a given assay was generally between 5 and 10 (so, we used a p value 10-fold lower than 0.05). Second, all comparisons with Ci-WT are indicated by a black asterisk; other pairs, whether indicated by brackets or described in the legend, use red asterisks. Third, comparisons with higher p values are not indicated on the graphs, but the p values are in supplementary spreadsheets to confirm the comparison was made and the outcome.

So, for Fig. 1N (previously M), only differences to Ci-WT are shown because that is the key issue. For Fig. 1O, red asterisks show significant differences to Ci-Δ1370-1397 because the key issue is whether Ci variants have lower Ci-155, similar to this Ci variant with a deficient direct Su(fu) interaction. The supplementary spreadsheet shows the significance of differences to Ci-WT.

4. For all the figures, the authors should label the staining with colored text to make it easier to follow.

This has now been done using the shortest acronyms- Plz, Ci, En

5. Figure 3, if the authors want to use Figure 3G-H to compare the levels of Ptc-lacZ and Ci155, they need to show the density data of Ptc-lacZ and Ci155 at the border. 

We believe the reviewer is asking for details of calculations and full transparency of all measurements. We believe that both were already in the manuscript but the comment suggests that further clarification is required even for an expert reader. We therefore have made the following adjustments for this and other figures.

(a) We have added to and edited the description of quantitation in Methods (current lines 1131-1209).

(b) We have added a reference to the Methods and the relevant supplementary data spreadsheets in each figure legend and in the main text at the first mention of clone and AP border measurements (current lines 232-6).

(c) For the specific issue cited here, we believe that the key point to clarify is that clone measurements are compared to the AP border of control discs. Hence, AP border intensities for experimental discs are not used in calculating clone values. We nevertheless did measure experimental disc AP border values and we include those data in supplementary spreadsheets.

6. A Ptc-luciferase assay would be more sensitive compared to the wing disc. The authors would have used some cultured cells for such an assay.
We agree with the reviewer that there are ways to obtain more accurate or sensitive readouts of *ptc* promoter activation. However, the central concept in our work is that Ci activity must be investigated under physiological conditions in a normal *in vivo* setting. We are grateful that editorial comments are in agreement, while also affirming and accentuating the reviewer’s insistence on full and careful documentation and analysis of the quantitative in vivo data.

7. How did the authors compare Ptc-lacZ and Ci expression across various wing discs? They used two Zeiss confocal microscopes, possibly using the same software and conditions for image acquisition. How were imaging conditions normalized?  Were laser intensity and other parameters normalized?
The response is largely outlined in response to point #5. The alteration in Methods text includes clarification of laser settings.

8. Line 642-643 (now 552-3) mentions 77% and 66%. How can readers judge these numbers if they are not shown in the figure? Although some information can be dig out from supplemental files, it is not easily comprehensible. This issue applies to all such figures.

We had originally excluded a small number of results from graphs and images. We have now included all cited data in graphs and images. For these specific data we have added the information for wing discs with one copy of the Ci variant gene to Fig. S4 (which previously showed only 2-copy data for the same variants).

We hope that the changes outlined in answer to point #5 make it easy to understand the supplementary spreadsheets, where to find them and how the raw data were processed.

9. Regarding Ci phosphorylation by Fu, although the dose-dependent model is well accepted, the data presented in this study does not exclude the possibility of sequential phosphorylation. Does combining CiA sites with CiD sites change Ci activity? These experiments can be tedious because of the many sites to consider; however, the authors should at least discuss this possibility.

The recent paper from Dr. Jiang’s lab examining how Fu gains access to Ci within Ci-Su(fu) complexes is now discussed in the Discussion. Specifically, those results would fit with a suggestion that phosphorylation of one site may make other sites more accessible (outlined in the opening section and the ordered phosphorylation section).

Minor concerns:

10. Figure 1E-E'', explain why some of the cos2 clones exhibit higher levels of Ci staining by the 2A1 antibody.
(Now 1F-F”) The variations in intensity are largely due to folds in the wing disc. You can see, for example, in this sample that plz and Ci-155 show similar variation. For quantitation, we avoid clones that include folds. Most scored clones are in the wing pouch, which generally has no folds.

11. Line 192 (now 195; response 206-9), what about Fu null mutant or using a dominant-negative Fu?

We added a sentence at the end of the paragraph to explain that complete loss of Fu gives strong loss of plz but Fu also has a non-catalytic function to promote Ci processing, so that Ci-155 is elevated throughout the disc. Hence, the choice of mH63 to investigate only the effects of Fu kinase activity.

12. Figure 1 D-D', needs a higher magnification to show Ci levels near the border.

We added a high magnification image pair as E-E’. Ci-155 profiles at the AP border for several genotypes, including loss of Su(fu) were shown in and discussed in detail in Little et al (2020).

13. Figure1, for all figures indicating Ci variants, the authors may want to use crCi or crCixxx to indicate that this form of Ci is not an overexpression, similar to the name "gCos2".

This is a good suggestion but we chose not to do this for two reasons- (i) it would take up space in labels that are already often occupying all space available & would therefore reduce clarity and (ii) the text makes clear that a key aspect of the study is using CRISPR alleles & we do not ever use other forms of Ci (when Su(fu) is expressed we use UAS-, and we use gCos2 to distinguish different types of transgenes), so it should be clear that Ci variants always refer to CRISPR replacements. 

14. Line 273-274, the statement "…suggesting that the higher Ci levels in the first three variants report greater Ci stability" is confusing, based on the data shown in Figure 1M and Figure2J.
We amended this to simplify and to use explicit Ci names throughout, making comparisons only to del1370-97.

15. Figure 2A", the image was out of focus. 
We believe the focus is normal and that there may have been confusion about what is shown. We therefore further clarified in the legend that this is showing a zoomed in version of A, where the levels of signal are very low.

16. Figure 2E-E', this disc is significantly larger. Is that because of the deletion of 175-230? Explain. 
We have added a potential explanation to the legend. For this & other larger discs we think it is likely due to loss of repressor activity & dpp induction.

17. Line 294-298 (revised version 267-275), confusing statement.
We have re-written this to make clearer that we are considering the potential contribution of En induction and repression of ci transcription in *cos2* clones. This is excluded as a reason for high Ci-155 levels for variants like del270-300 on the basis that En induction is lower than for del1370-97. At the AP border of fu mutant discs there is no AP border En induction and this is not a factor.

18. Line 300 (now 280), "interface" or "interaction"?
We have left this as “interface”. We believe these terms are essentially equivalent, except that “interface” more obviously refers to a direct interaction, which we are trying to emphasize here.

19. Line 311-312, what "clones"? How did the authors measure Smo activity in "anterior clones"?

We believe there is some misunderstanding here. We changed the text to say “we created clones that expressed UAS-GAPFu”. We then explain that the clones genetically lack *smo* activity (lines 304-7).

20. Line 317-320, the statement "The increase in Ci-155…..by GAP-Fu" is a confusing. 
We now clarify by saying “caused by expression of GAPFu” (lines 313-315).

21. Figure 3, how were the 42% and 30% calculated? 
The methods and data for all such clone calculations were clarified in answer to point #5.

22. The ci allele with Ci-Sufu fusion is a smart design. Is Ci processing changed by fusing Sufu to the C-tail of Ci. Although their lab might not use cultured cells, Ci processing can also be detected using wing disc samples.
The pattern of Ci-155 elevation at the AP border and in cos2 clones shows there is robust Cos2-dependent processing that is inhibited by Hh. Ci-75 per se is not detected but it is likely that its absence would lead to enlarged discs (not seen).

23. Do the authors have a control for Ci-Sufu, e.g., adding another sequence to Ci C-tail? 
We did not test addition of unrelated “random” sequences or protein domains. However, a key control for inhibition relying specifically on the nature of the Su(fu) moiety was investigation of the same construct with the SYAAD alteration. This substantially changed Ci activity in our assays, showing that at least some of the reduction in activity due to adding Su(fu) was due to specific Ci-Su(fu) interaction.

24. How does the 1:1 Ci:Sufu ration hypothesis apply to anterior cells far away from the AP border?
The activity of Ci-WT-Sufu was low in cos2 clones throughout the wing pouch and the rest of the disc, so the conclusion that one Su(fu) per Ci suffices for inhibition applies throughout the wing disc.

25. Figure 4, Ci-WT-Sufu equals to Ci-SuFu, correct?
Yes, we have made alterations to always call this Ci-WT-Sufu now.

26. Line 478 (now line 423), Ci-delta-delta has a slightly greater activity deficit than Ci-A1A2. Does this mean the deleted amino acids have additional positive roles in regulating Ci? How to further confirm this?
The differences in activity in GAP-Fu clones and at the AP border are small. It is possible that the deletion compromises activation in additional ways but investigating defects due to a deletion is always difficult, so we prefer not to make a significant issue of the differences.

27. The use "SuFu" needs to be consistent throughout the manuscript. The authors can possibly use "dSufu" for fly and "mSufu" for mouse. 
We did find it difficult to deal with the different conventions used in Drosophila and mammalian work. We are dealing mostly with Drosophila and yet we also wish to make general statements about Hh signaling that includes vertebrates. We settled on a compromise that is explained in lines 96-97, respecting the usual conventions used for each organism but using “Sufu” to refer to the generic case. When specifically using a mouse gene in our experiments we write “mSufu”. Everything else experimental is with Drosophila genes, so that Su(fu) is relevant. We think that this will be clear.

28. Line 568 (now line 514) "Fu-EE, which has acidic residues…" should be described when Fu-EE first appears. 
We have now put the explanation for Fu-EE (& for GAP-Fu) in line 118-122

29. Line 597 (now line 524), define the name "gCos-AA"
We have added gCos-WT and gCosAA to the previous sentence, where they are explained.

30. Figure 7, Needs to show Ptc-lacZ to mark the border. 
We have added Ptc-lacZ in red to images showing the GFP-marked clones.

31. Figure 8A', Ci is very bright with normal staining pattern. How the authors standardize the procedure for taking images?
We have explained how images are used for quantitation in response to point #5. We usually show images with similar processing. However, if the staining is very low for some genotypes, the image shown has increased brightness and the comparison samples have a similar adjustment, so the reader sees an appropriate comparison (importantly, this choice of image presentation does not affect quantitation). Fig. 8A’ is indeed too bright and was a mistake- we have now normalized this in the usual way.

32. Figure 8 K-O", remove the yellow lines on the top. Those are not the focus.
Done

33. Figure S1B, what about cos2 clones away from the AP border? Also in S1B legend, "second line shows deletions…..binding domain" needs clarification.
We did not perform En staining on many samples and do not have any examples of anterior clones further from the AP border. It is conceivable that small amounts of Hh reach the clone illustrated, but as shown by the ptc-lacZ pattern, the clone is anterior to the AP border. Thus, we believe that En induction is independent of Hh signal.

We have replaced “line” in each case with “cartoon”.

Summary

We are very grateful for the reviewer’s careful attention to detail and appreciation of the major findings. We understand that the major deficit for the reviewer, on behalf of all readers, is the lack of direct Ci-Ci molecular interaction data. As explained earlier (point #1), this would be a major undertaking and we hope the reviewer is satisfied that the arguments we present constitute a strong basis for a model that will inevitably be tested in detail in future. We have used the reviewer’s extensive comments to improve the clarity and presentation of the manuscript, hopefully answering each specific question and considering each suggestion.

COMMENTS FROM THE ACADEMIC EDITOR

The relationship between Fu kinase, Su(Fu), Cos2, and Ci transcription factors during Hedgehog (Hh) signal transduction has remained unresolved. This question has been largely investigated in cultured cells using overexpressed proteins - led to numerous propositions, but to what degree they apply to endogenous Hh signaling under physiological conditions is unclear. In this study, Kim et al. utilize a Drosophila wing imaginal disc model, a well-established system for Hh signaling, to address this gap by generating CRISPR-based mutations in endogenous pathway components, thereby avoiding some of the pitfalls of prior overexpression strategies. This approach allowed them to evaluate various mutations in Ci, which are implicated in Fu phosphorylation, Su(Fu) binding, or both.

Principal Findings

-Multiple regions on Ci, including areas around previously defined Su(Fu) binding sites, contribute to Ci activation.
-Fu's activation of Ci does not strictly require Su(Fu) binding or Cos2 interaction. This supports the idea that Fu phosphorylation enables Ci to adopt an activated conformation, rather than merely escaping Su(Fu) inhibition.
-Fu phosphorylation of Cos2 and Su(Fu) is largely dispensable for Ci activation.
-The identified Fu phosphorylation sites do not fully explain Fu's action on Ci, implying the existence of additional phosphorylation sites that have not yet been mapped.

Whilst some of these findings have been reported earlier, the present study adds value by validating them using endogenous pathway components in a physiological system.


Recommendation: Support publication in PLOS Biology pending the following revisions:

-Quantification of Figures: Quantification is missing from several key figures. For example, the deletions of residues 301-320 and 321-346 in Fig. 1 are not quantified, and the text refers to deletions of residues 175-232, while the figure panel mentions 175-230. Additionally, figures 5 and 8 lack any quantification. These omissions should be addressed to ensure the results are properly substantiated.

The general point about quantitation and presentation of the significance of difference between values is addressed in response to reviewer point #5.

For the specific items mentioned here, we have added data for Ci variants lacking 301-320 & 321-346 to the bar graphs.

We have corrected 175-230 in the text.

Figs. 5 and 8 were (previously) fully quantitated. As part of the response to reviewer point #5 we have added more information about where to find quantitation. These are referenced in the legend to Fig. 5 (the graphs & supporting files are found elsewhere) and similarly for Fig. 8.

The reason for putting some graphical data in other locations is because we describe successive steps in our investigation in the text but, in the end, we would like to compare all Ci variants subjected to a specific assay. Thus, the graphs collect together Ci variants, which are sometimes initially described in more than one Figure. The graph is therefore necessarily sometimes in a different Figure to the images presented for specific variants.

-Streamlining the Discussion: The Discussion is overly long and difficult to follow, particularly for non-experts. I recommend simplifying it by adding a concise summary paragraph near the beginning that highlights the principal findings and their conceptual significance. I also suggest trimming redundant sections for clarity.

We retain the first paragraph of the Discussion to re-iterate the key feature of this work- examination of Hh signaling under physiological conditions. We add a sentence at the end that summarizes the most important deduction we have made.

The next section explains the most important deduction and underlying evidence. We have shortened and clarified the previous text in this section. We have also added a final paragraph that explains the major findings of a recent publication concerning Fu kinase access to Ci-Su(fu) and draw attention to the complementary nature of our findings (later we refer to that paper again for two specific hypotheses).

We believe that most readers will find the two sections mentioned sufficient for understanding our biggest conclusion in context.

We have also edited subsequent sections to incorporate insights from the recent paper mentioned above.

We would like to retain these sections. We believe each section topic is important and grouping by these topics makes the Discussion significantly different from Results sections, affording a more critical and comprehensive appraisal. This is partly for the usual reason of being able to draw together various results presented at different locations in the Results (as the topic moves with steps in the investigation). It is also because some of the logic is quite complicated. We have edited these sections for greater clarity and deleted some segments (but not drastically). We suspect that these sections will be very useful to readers seeking to extract maximum benefit, with more careful consideration of both details and the evidence supporting our major conclusion. We found that writing and editing these Discussion sections was important for refining our own conceptions and arguments and we deliberately simplified presentation of some conclusions in the Results in anticipation of further clarification at a more optimal time in the Discussion.

-Structural Model in Figure 9: While the data generally support the model that Ci undergoes a conformational change, the structural model presented in Fig. 9 is difficult to follow and reads as highly speculative, because it is largely based on inferences from mutational analysis rather than direct biophysical or structural data, and no three-dimensional structural model of Ci is available. These limitations should be more clearly acknowledged in the Discussion.

We agree that the model is based on genetic inference without direct protein interaction or structural evidence and we agree with the objective of making that clear. We have already done that to a good degree but have made further edits. As noted, the legend accompanying an explicit model (or range of models) in Fig. 9 “reads as highly speculative”. We have altered the first sentence to make even clearer that this is one rendition of a range of speculative general models.

Two sentences were added to the first paragraph of the Discussion to make clear that our inferences are from genetic data alone, that biochemical tests will be essential to confirm our model and uncover further detail, and that Fig. 9 illustrates one specific speculative model.

“It will be important to test our genetic inferences by identifying specific Ci-Ci interfaces directly within Ci-Su(fu) complexes of various activity states. We nevertheless illustrate our deductions and speculations about Ci activation mechanisms in the discussions below with reference to a plausible specific model (Fig. 9).”

Most of the relevant Discussion is in the first titled sub-section. It already had several “perhaps”, “potentially” and so forth indicating uncertainties (one or two added). Most important, the key section, which has now been abbreviated, hopefully amplifying the central point, cites the explicit evidence underlying the main proposal. The gap between the evidence, the general surmise, and one specific set of models presented in Fig. 9 should be clear. In the next section, where specific phosphorylation sites are discussed, the reasoning behind choosing to show specific speculative Ci-Ci interactions is explained with the phrase “It is even possible that the partially additive and partially synergistic effects of S218A and S1230A alterations reflects a direct interaction between these two regions of Ci that is altered by phosphorylation (Fig. 9).” That should make clear that the specific interactions shown are not being claimed as backed by solid evidence.

-Clarification of GAP-Fu: The manuscript refers to GAP-Fu, which, according to the referenced literature, is a membrane-targeted Fu construct. This should be clearly defined within the text to avoid confusion for readers.
This explanation was expanded at first mention (line 118-122) together with explanation of Fu-EE.

With these relatively minor revisions, I believe the manuscript will significantly contribute to the field by clarifying the endogenous roles of Fu, Su(Fu), and Cos2 in Hh signal transduction.

Summary

We are very grateful for the editorial reviewer’s appreciation of the value of our findings and approach, even acknowledging that more detailed molecular and structural insights will be required at some stage to verify and refine our conclusions. One major issue to address concerned quantitation. We have clarified where those data can be found, added some images and graphical data, so that all quoted data are presented equally, clarified the methods for quantitation and added measures of the significance of differences in values. We believe that issue is comprehensively addressed. The second issue related to clarity of distinctions between conclusions and speculations, together with cleaner emphasis of key findings in the discussion. We believe the speculation/conclusion issue is clear in the text. We hope that modifications to the untitled section and the first titled sub-section of the Discussion now serve the requested purpose of emphasizing the main conclusions, and that it is a legitimate expectation that some readers may wish to stop there. One reason for selecting submission to this journal was the lack of specific page limits, allowing more potential for full explanations. I hope the editors and interested readers will agree with our opinion that the remaining sections of the Discussion are interesting and important for gathering strands together in a tricky ongoing complex puzzle, with placement at the end minimizing the chance of distracting readers from the main conclusions.
